# Abdominal wall hernia management in a second level hospital of Senegal: a cross-sectional study

**DOI:** 10.3389/fsurg.2026.1864822

**Published:** 2026-06-26

**Authors:** Guillaume Tcheutchoua Soh, Seneba Aicha Gaye, Papa Mamadou Faye, Thierno Amadou Telly Diallo, Abdoul Kharim Diop, Jacques Noel Tendeng, Ousmane Thiam, Philippe Manyacka Ma Nyemb, Alpha Oumar Toure, Ibrahima Konate, Mamadou Cisse

**Affiliations:** 1 Department of Surgery, Gaston Berger University, Saint-Louis, Senegal; 2 Department of Surgery, St Christopher University, Dakar, Senegal; 3 Department of Surgery, Universite Cheikh Anta Diop Bibliotheque Universitaire, Dakar, Senegal

**Keywords:** Africa, cross-sectional study, hernia, low- and middle-income countries, semi-urban

## Abstract

**Introduction:**

Hernias represent a common pathology worldwide. In low-income settings, there is difficulty in implementing existing guidelines, and a lack of research funding prevents the development of local guidelines. This article aims to describe the current management of abdominal wall hernias in a secondary centre in Senegal.

**Method:**

We conducted a cross-sectional study over a six-month period in the surgical department of a second-level centre hospital based in a semi-urban area of Senegal.

**Results:**

Among 199 surgeries performed at our centre during this period, hernia repairs accounted for 34.2% of surgical activity. Inguinal hernias accounted for 25.6% and midline ventral hernias for 8.5% of all procedures, with inguinal hernias constituting 75% of the hernias operated on. All inguinal hernias were primary, and 82.3% were primary midline ventral hernias. Procedures performed on an emergency basis were 19.1% of repair. A mesh was used for 51.5% of hernias and tissue repair for 48.5%. Treatment of inguinal hernias was performed under spinal anaesthesia in 90.2% of cases and under general anaesthesia in 9.8% of cases. The treatment techniques used were Lichtenstein in 64.8% of cases, Desarda in 33.3% of cases and Bassini in 2% of cases. Complications included pain at the surgical site in 17.7%, lower urinary tract obstructive disorders in 11.8% including acute urinary retention in 5.9%; headache in 7.8% and spermatic cord induration in 9.8%. Treatment of midline ventral hernias was performed under general anaesthesia in 100% of cases. This involved Primary aponeurotic closure in 88.2% of cases and Onlay repair in 17.7 of cases. Complications included pain at the surgical site in 5.9%, infection at the surgical site in 5.9% and post-operative ileus in 5.9%.

**Conclusion:**

The management of abdominal wall hernias has advanced in our region. However, efforts are needed to raise awareness among the population and encourage consultation before complications arise. Complications related to spinal anaesthesia are numerous. Guidelines adapted to Africa need to be developed in order to standardise the treatment and follow-up of patients with abdominal wall hernias.

## Introduction

Abdominal wall hernias are a common condition worldwide and significant technical advances have been achieved in their management. Most contemporary surgical techniques rely on mesh reinforcement of the abdominal wall. Numerous recommendations have been made by scientific societies such as the European Hernia Society and the American Hernia Society (1). There is broad consensus on the importance of offering individualized treatment tailored to each patient, taking into account hernia risk factors, clinical characteristics, surgical risk, and the risk of recurrence (1–3).

In low-income settings, particularly in Sub-Saharan Africa, hernia are a particular concern; they are common disease with exact prevalence unknown, some risk factor like heavy work and high maternity are particularly present in Sub-Saharan Africa (4, 5). Recent systematic review conducted by Ndong et al. reported that Bassini (40,1% of technique used), an older modern technic of inguinal hernia repair are still commonly used in Africa despite it is criticized for his high prevalence of recurrence (6, 7). And Lichtenstein (the gold standard) is used in 29.9% of inguinal hernia reparation (6). Most ventral hernia repairs are done with tissue repair and preoperative optimisation is rarely applied (3, 8). The implementation of existing guidelines remains challenging despite development of some tips like use of low-cost mesh or emerging of cure without tension like Desarda, popular in LMIC (9, 10).

In Senegal, data on hernia are limited. The prevalence of hernia in adult is unknown. As in other areas of sub-Saharan countries, low access to surgery contributes to observation of high rates of strangulated hernias and giant hernias (3, 9, 11). Furthermore, there is a rarity of long-terms outcomes after hernia repair. For example, studies conducted by Ndong, Tendeng and Ngom had follow-up periods of 18.2 months, 15 months and 14 months (3, 9, 12). This is particularly important for recurrence rates and quality of life after hernia repair, which have an epidemiology different depending of the follow-op duration. Three to five years of follow-up is generally accepted (13). A recent study also demonstrates a knowledge gap regarding chronic pain prevention and management in our area (14). Moreover, limited research funding hinders the development of context-specific recommendations for the best approach for hernia management. As a result, patient management often depends on the surgeon's expertise and the resources available. This article aims to describe the management of abdominal wall hernias in a second level centre in Senegal and to compare our practice with available data in order to explore potential areas for improvement for our practice.

## Patients and methods

### Study design

This was a cross-sectional study conducted over a six-month period in a second level hospital in a semi-urban area of Senegal. The work was carried out in accordance with the STROBE guidelines (15).

### Inclusion criteria

It included all patients (>15 years) admitted to the general surgery ward for treatment of an abdominal wall hernia, emergency and elective.

### Exclusion criteria

We excluded patients whose records were incomplete and a lack of follow-up. Patients who refused to participate in the study.

### Operator details

All patients were operated by 2 general surgeons with more than 2 years of independent practice. The surgical technique was chosen by the surgeon who followed the patient or received the patient, in case of emergency admission.

### Ethical approval and patient consent

The study was approved by the hospital's institutional ethics committee. Verbal consent was obtained from each patient before their inclusion in the study. Each patient's data was anonymised before inclusion in the study in order to remove any information that could identify the patients.

### Data collection

Data were prospectively collected from patient records. According to our procedure, data were collected during the follow-up during hospitalisation, at 1 week and 1 month. In case of complication, the patient was assessed as required.

### Variables studied

Variables were age, gender, risk factors for hernia: heavy labour (worker in a high-intensity job), active smoker, obesity (BMI > 30), constipation, lower urinary tract obstructive disorders, chronic cough; clinical presentation, types of hernia (inguinal and ventral hernia) and associated abnormalities, anaesthesia and surgical techniques, and their early results.

### Data management and analysis

Each patient's data was entered into a form using Microsoft Excel 2016 software. The data was analysed using SPSS 25 software. Qualitative variables were expressed as frequencies and percentages. Quantitative variables were expressed as mean, standard deviation, median, maximum and minimum.

## Results

### Epidemiology

During the study period, a total of 199 surgeries were performed at our centre, including 68 surgeries for abdominal wall hernias, representing 34.2% of the surgeries performed. Inguinal hernias accounted for 25.6% and ventral hernias (non-inguinal) for 8.5% of our surgical activity. Inguinal hernias, commonest type of hernias, accounted for 75% of hernia operations. There were a male predominance 76.5% (*n* = 52) and the females reprensented 23.5% (*n* = 16) of patients. The sex ratio was 3.25. All inguinal hernias were primary, and 82.3% were primary midline ventral hernias. The most common risk factors were heavy manual labour (35.3%), lower urinary tract obstructive disorders (26.5%), followed by constipation (20.6%) multiples pregnancies (16.2%) and Smoking (16.2%). Procedures performed on an emergency basis were 19.1% of case. Epidemiology, Patient features, risk factors are summarised in Tables 1, 2.

**Table 1:** Patients features.

Variables	Inguinal hernia *n* = 51	Ventral hernia *n* = 17	Total *n* = 68
Age (mean ± SD)	56.01 ± 18.2	45.05 ± 12.7	53.23 ± 17.58
Sex			
Men	49 (96.08)	3 (17.7)	52 (76.5)
Women	2 (3.9)	14 (82.4)	16 (23.5)
History of pain	33.3 (17)	0	17 (25.0)
Clinical Presentation			
Emergency	11 (21.57)	2 (11.8)	13 (19.1)
Elective	40 (78.43)	15 (88.2)	55 (80.9)
Duration of symptoms (emergency case) in hour (mean ± SD)	22.36 ± 14.34	120	37.38 ± 39.93

**Table 2:** Patien's risk factors.

Risk factors	Inguinal hernia *n* = 51	Ventral hernia *n* = 17	Total
Heavy manual labour	23 (45.1)	1 (5.9)	24 (35.3)
Chronic cough	7 (13.7)	0 (0)	7 (10.3)
Obesity	0 (0)	10 (58.9)	10 (14.7)
Constipation	13 (25.5)	1 (5.9)	14 (20.6)
Multiple pregnancies	2 (3.9)	9 (52.9)	11 (16.2)
Smoking	11 (21.6)	0	11 (16.2)
Lower urinary tract obstructive disorders	18 (35.3)	0	18 (26.5)
Benign prostatic hypertrophy	4(7.8)	0	5(7.4)

### Treatment and outcomes

A mesh was used for 51.5% of hernias and tissue repair for 48.5%. Treatments and outcomes are summarised in Table 3 and Table 4.

Treatment of inguinal hernias was performed under spinal anaesthesia in 90.2% of cases and under general anaesthesia in 9.8% of cases. The treatment techniques used were Lichtenstein in 64.8% of cases, Desarda in 33.3% of cases and Bassini in 2% of cases (Table 3). Complications included pain at the surgical site in 17.7%, lower urinary tract obstructive disorders in 11.8% including acute urinary retention in 5.9%; headache in 7.8% and cord induration in 9.8% (Table 4).

**Table 3:** Data of treatment.

Variables	Inguinal hernia *n* = 51	Ventral Hernia *n* = 17	Total
Hernia location			
Indirect	38 (74.5)		38 (55.9)
Direct	13 (25.4)		13 (19.1)
Femoral	0		0
Umbilical		6 (35.3)	6 (8.8)
Epigastric		7 (41.2)	7 (10.3)
Sub-umbilical		3 (17.7)	3 (4.4)
Type of hernia			
Primary	51 (100)	14 (82.4)	65 (95.6)
Secondary	0	3 (17.7)	3 (4.4)
Type of anaesthesia			
Spinal	46 (90.2)	0	46 (67.7)
General + orotracheal intubation	5 (9.8)	17 (100)	22 (32.4)
Associated Anomalies	6 (11.8)		6 (8.8)
Ectopic testis	1 (2)		1 (1.5)
Persistence Processus vaginalis	2 (3.9)		2 (3)
Hydrocele	1 (2)		1 (1.5)
Amyand hernia	1 (2)		1 (1.5)
Richter hernia	1 (2)		1 (1.5)
Type of repair			
Tissue	18 (35.3)	15 (88.2)	33 (48.5)
Desarda	17 (33.3)		
Bassini	1 (2)		
Primary aponeurotic closure		15 (88.2)	
Prosthetic	33 (64.8)	2 (11.8)	35 (51.5)
Lichtenstein	33 (64.8)		
Onlay repair		3 (17.7)	
Length of hospital stay in day (mean ± SD)	1.16 ± 0.55	1.76 ± 1.56	1.31 ± 0.94

**Table 4:** patients outcomes.

Post-operative complications	Inguinal hernia *n* = 51	Midline ventral Hernia *n* = 17
Post-operative pain	9 (17.7)	1 (5.9)
Headache	4 (7.8)	0
Obstructive urinary tract symptoms	6 (11.8)	0
Acute urinary retention	3 (5.9)	0
Cord induration	5 (9.8)	0
Surgical site Infection	0	1 (5.9)
Ileus	1 (2)	1 (5.9)
Death	1 (2)	0
Clavien-Dindo		
Grade I	10 (19.7)	1 (5.9)
Grade II	15 (29.4)	2 (11.8)
Grade III	0	0
Grade IV	0	0
Grade V	1(2)	0

Treatment of ventral hernias was performed under general anaesthesia in 100% of cases. This involved Primary aponeurotic closure in 88.2% of cases and Onlay repair in 11.8% of cases (Table 3). Complications included pain at the surgical site in 5.9%, infection at the surgical site in 5.9% and post-operative ileus in 5.9%.

Grade II complications were the most common (29.4%) for inguinal hernias and 11.8% for ventral hernias (Table 4). The death case identified in our study was a 46-year-old patient with psychiatric disorders who was not receiving regular medical care. He presented with a left inguinal hernia that had been strangulated for two days, signs of peritoneal irritation and signs of severe sepsis. Following brief resuscitation, he underwent surgery via a laparotomy combined with an inguinal incision. The hernia sac contained 20 cm of necrotic ileal loops and approximately 200 mL of pus. We resected the necrotic bowel and performed a double-barrel ileostomy, carried out a repair according to the Desarda method, and performed abdominal washing and drainage. The patient died in the immediate post-operative period, four hours after admission to the intensive care unit.

## Discussion

### Epidemiology

Hernia repair constitutes a major part of surgical activity in Africa. In our study, it accounted for approximately 34.2% of all procedures during the study period. These results are similar to those reported in studies conducted in Benin and Senegal (16). Kefleysus et al. identified hernia repair as the most frequently performed procedure in general surgery (17).

The risk factors found, in order of frequency, were heavy manual labour, lower urinary tract disorders, constipation, multiple pregnancies and obesity, which are well known in the medical literature. Inguinal hernias represented the majority (approximately 75%) of wall hernias in our centre. This is the most common type of abdominal wall hernia (18). Inguinal hernias predominantly affect young men aged between 20 and 60 years, whereas midline ventral hernias are more frequently observed in women (1). In our centre, men were also more likely to have inguinal hernias and women were more likely to have midline ventral hernias. Among female patients, the higher prevalence of midline ventral hernias appears to be related to abdominal morphology, obesity, and especially repeated pregnancies. Similar findings were reported by Abdourahmane Ndong et al. in a study conducted in a second level centre in Senegal. In their series, obesity and multiple pregnancies were present in 58.9% and 53% of patients with midline ventral hernias, respectively (3).

### Clinical presentation

In our centre, 25% of patients reported experiencing acute painful episodes of hernia before consultation, and 19.1% of patients presented with strangulation. In Africa, the rate of hernia strangulation may reach up to 58.5% according to some African studies (16, 19, 20). This has also been confirmed by Ndong's systematic review (6). This delay can be explained by our socio-cultural context, where hernias are perceived as a benign and non-disabling condition (21). As a result, most patients only consult a doctor when they experience alarming symptoms such as pain. Conversely, in Europe, recommendations call for surgery for all diagnosed hernias, thereby reducing the rate of hernia strangulation (1). Regarding midline ventral hernias, 11.8% of patients in our study presented with strangulation.

### Treatment

#### Preoperative preparation and management of risk factors

The management of wall hernias begins with the management of modifiable risk factors, such as smoking cessation, weight loss or treatment of chronic constipation (1, 2). In our study, none of these risk factors were managed for either inguinal or midline ventral hernias. This should allow treatment to be tailored to each patient, as recommended by the European Hernia Society (1). In addition, 19.1% of our patients were admitted as emergency cases and therefore could not benefit from preoperative risk factor optimization, which may have negatively impacted our outcomes. Certain risk factors, when identified before surgery, can be managed during the same operation, for example, by performing a prostate adenectomy or TURP in conjunction with hernia repair (22). This reduce the number of hospitalisation, the cost of the treatment and exposure to anaesthesia without increase morbidity and mortality (23–25). This benefit has been reported in our context, but in older adults who underwent tissue repair, as part of a small-scale cohort study that lacked long-term results (26). We believe that the implementation of a standardised approach such as the use of structured surveys would facilitate the systematic identification of risk factors and allow appropriate optimization before surgery or targeted follow-up in the postoperative period.

### Inguinal hernia

#### Anaesthesia

In our study, spinal anaesthesia was the most commonly used anaesthesia technique accounting for 67.7% of cases (16, 22). However, international guidelines proposed by the European Hernia Society recommend the use of local anaesthesia as the first-line treatment or general anaesthesia (1,1). They base this recommendation on the fact that immediate post-operative complications are more common with spinal anaesthesia. This was the case in our study, where 11.8% of patients experienced lower urinary tract disorders and 5.9% experienced acute urinary retention. Other complications related to spinal anaesthesia were also noted, such as headaches(7.8%). Spinal anaesthesia is also less comfortable for the patient, as patients must remain lying flat for several hours, and in the event of urinary disorders, a urinary catheter may be required. Osifo and Olaogun demonstrated the feasibility of local anaesthesia for strangulated and giant hernias and that even in cases of necrosis, resection was possible (27, 28). Endoscopic treatment techniques require general anaesthesia but are not available at our centre. A meta-analysis by Argo et al. reported comparable outcomes between local and general anaesthesia (29). Local anaesthesia therefore seems to be the most suitable technique for our centre as it is accessible, has low morbidity, is cost-effective and may reduce length of hospital stay. However, adequate training of surgical teams is essential to ensure the safe and effective implementation of this technique. It has been reported training is one of the key factors which influencing the choice of surgical technique for hernia repair among residents in Senegal (30). Repair of a hernia under local anaesthesia is not taught in Senegal during the residency program. It has been reported that in Ghana, it was more frequently chosen in overseas-trained doctors as method of anaesthesia than locally trained doctors. It has a potential benefit when combined with outpatient surgery in sub-Saharan countries (31). It can be used in up to 52.3% of inguinal hernia repairs with low complication rate (32).

### Surgical technique

The Lichtenstein tension-free repair remains the gold standard for inguinal hernia surgery and was the most frequently performed technique in our centre, accounting for 64.8% of cases. This proportion is higher than that reported in earlier studies conducted in similar centres in Senegal approximately two decades ago, where the Lichtenstein technique represented only 29.9% of inguinal hernia repairs (6). This finding is consistent across Africa (10). In Ndong's systematic review, the most commonly used hernia repair technique in Africa between 2000 and 2020 was the Bassini repair (6). Our results suggest improved accessibility to prosthetic materials in our region (10). The external oblique aponeuroplasty repair described by Mohan Desarda, which has become popular in low- and middle-income countries, is the second most commonly used technique in our study (17). It has the advantage of avoiding the cost of purchasing a prosthesis, can potentially be used in all circumstances (even in cases of site infection), can be performed under local anaesthesia and, finally, its short- and long-term results are comparable to the current gold standard (33–35).

### Midline ventral hernia

#### Anaesthesia

The choice of anaesthesia for the repair of umbilical and epigastric hernias depends on the size of the defect, the surgical context and the resources available. Local anaesthesia is indicated for small, simple hernias (< 2 cm), particularly in outpatient surgery (36). General anaesthesia is reserved for large, recurrent or complicated hernias, as well as for extensive prosthetic repairs or emergency procedures (2). According to the recommendations of the European Hernia Society (EHS), the choice should be individualised, taking into account patient comfort, the type of hernia and the technical context of the operating theatre (2). General anaesthesia was used in our centre for all patients with midline ventral hernias.

#### Surgical technique

In our series, suture repair was performed in 88.2% of patients with midline ventral hernias, while prosthetic repair was used in 17.7% of cases, corresponding to three patients with secondary midline ventral hernias. The rational choice of technique for a midline ventral hernia depends on the size of the hernia neck, whether it is primary or secondary, the risk of recurrence, the risk of infection and the patient's condition (2). In our study, the only characteristic taken into account was whether the hernia was primary or secondary.

Early postoperative outcomes demonstrated low morbidity, consisting mainly of surgical site infections with favourable outcome under antibiotic therapy and local wound care, immediate postoperative pain in one patient, and postoperative ileus that resolved spontaneously without specific treatment. However, the duration of follow-up in our study was insufficient to assess chronic pain, recurrence and quality of life after surgery. We believe that longer term studies are necessary to better evaluate these outcomes.

### Limitations and prospects

At the end of our study, we note the following limitations: a small sample size, a short follow-up period, the aim of our study is purely descriptive; it was intended much more to describe the management of the condition than to investigate long-term outcomes. It should be noted that, as a rule, the lack of insurance means that the cost of consultations is borne by the patients, who often live a long way away. In our usual practice, provided there are no complications beyond the initial consultation, follow-up consultations are carried out at the patients' request. Also the fact that the study was conducted in a single centre, which limits the evaluation of long-term results and means that the results cannot be generalised to the entire region.

In addition, the study also highlights some potential solutions that could improve the management of abdominal wall hernias in our region. These include increasing public awareness of the importance of early surgical management of minimally symptomatic hernias, given the risk of complications; identifying and treating modifiable risk factors before surgery, where possible; promoting Desarda hernia repair under local anaesthesia as a potential treatment of choice, given the benefits of this approach in inguinal hernia; and finally, developing a standardised approach to better select treatments for midline ventral hernias, given the availability of prosthetic materials. This was also highlight by Basimbe Francis in his study (37).

## Conclusion

The management of abdominal wall hernias has seen significant advances in our region. However, efforts are still needed to raise awareness among the population and encourage consultation before complications arise. Complications related to spinal anaesthesia are numerous. Guidelines adapted to Africa need to be developed in order to standardise the treatment and follow-up of patients with abdominal wall hernias.

## Data Availability

The raw data supporting the conclusions of this article will be made available by the authors, without undue reservation.

## References

[1] HerniaSurge Group. International guidelines for groin hernia management. Hernia. (2018) 22(1):1–165. 10.1007/s10029-017-1668-x PMC5809582 29330835

[2] Henriksen NA Montgomery A Kaufmann R Berrevoet F East B Fischer J. Guidelines for treatment of umbilical and epigastric hernias from the European hernia society and americas hernia society. Br J Surg. (2020) 107(3):171–90. 10.1002/bjs.11489 31916607

[3] Ndong A Tendeng JN Soh GT Diallo AC Diao ML Sarr N. Management of midline ventral hernias in a surgical department of sub-saharan Africa: a retrospective cohort study. Ann Med Surg (Lond). (2022) 78:103801. 10.1016/j.amsu.2022.103801 35734700 PMC9206929

[4] Ajong AB Agbor VN Simo LP Noubiap JJ Njim T. Grand multiparity in rural Cameroon: prevalence and adverse maternal and fetal delivery outcomes. BMC Pregnancy Childbirth. (2019) 19:233. 10.1186/s12884-019-2370-z 31277596 PMC6612095

[5] Beard JH Oresanya LB Akoko L Mwanga A Dicker RA Harris HW. An estimation of inguinal hernia epidemiology adjusted for population age structure in Tanzania. Hernia. (2014) 18(2):289–95. 10.1007/s10029-013-1177-5 24241326

[6] Ndong A Tendeng JN Diallo AC Diao ML Sow O Mawuli SD. Adult groin hernia surgery in sub-saharan Africa: a 20-year systematic review and meta-analysis. Hernia. (2023) 27(1):157–72. 10.1007/s10029-022-02669-9 36066755

[7] Beets GL Oosterhuis KJ Go PM Baeten CG Kootstra G. Longterm followup (12–15 years) of a randomized controlled trial comparing bassini-stetten, shouldice, and high ligation with narrowing of the internal ring for primary inguinal hernia repair. J Am Coll Surg. (1997) 185(4):352–7. 9328383

[8] Ayandipo OO Afuwape OO Irabor DO Abdurrazzaaq AI. Adult abdominal wall hernia in ibadan. Ann Ib Postgrad Med. (2015) 13(2):94–9. 27162521 PMC4853882

[9] Tendeng J Ndong A Da D Diallo A Diop S Soh G. La cure auto-prothetique sans tension par lambeau de l'aponevrose Du grand oblique dans les hernies inguinales etranglees de l'adulte Au CHU de saint-louis du Senegal : à propos de 38 cas. Revue de Chirurgie d'Afrique Centrale (RECAC). (2021) 4:20–2021.

[10] Tabiri S Yenli EMT Gyamfi FE Jalali A Nelson RE Price RR. The use of mesh for inguinal hernia repair in northern Ghana. J Surg Res. (2018) 230:137–42. 10.1016/j.jss.2018.04.058 30100030

[11] Diop B Sall I Ba PA Konaté I Sy A Wane Y. Management of giant inguinal hernias: five cases. Med Sante Trop. (2013) 23(1):30–4. 10.1684/mst.2013.0133 23448670

[12] Ngom G Zeng FTA Sagna A Gueye D Ndoye NA Mbaye PA. Management of umbilical hernia in African children: the experience of 2146 cases. J Indian Assoc Pediatr Surg. (2023) 28(3):212–7. 10.4103/jiaps.jiaps_115_22 37389397 PMC10305955

[13] Plackett TP. Long Term Outcomes for Inguinal Hernia Repairs at a Military Treatment Facility—SAGES Abstract Archives. SAGES (2019). Available online at: https://www.sages.org/meetings/annual-meeting/abstracts-archive/long-term-outcomes-for-inguinal-hernia-repairs-at-a-military-treatment-facility/ (Accessed May 13, 2026)

[14] Ndong A Diallo AC Falola AF Ndiaye MA Faye M Faye PM. Knowledge and practices regarding chronic pain after groin hernia surgery: a national resident survey in Senegal. J Abdom Wall Surg. (2025) 4:13764. 10.3389/jaws.2025.13764 40151490 PMC11947562

[15] Cuschieri S. The STROBE guidelines. Saudi J Anaesth. (2019) 13(Suppl 1):S31–4. 10.4103/sja.SJA_543_18 30930717 PMC6398292

[16] Hodonou AM Sambo BT Gandaho IE Babatounde A Allodé AS Mensah E. Caractéristiques epidémiologiques et thérapeutiques des hernies pariétales au centre hospitalier universitaire du borgou A parakou, bénin. WWJMRD. (2018) 4:43–6.

[17] Kefleyesus A Demartines N Schäfer M Allemann P. Chirurgie des hernies de la paroi en 2018 : mise au point. Rev Med Suisse. (2018) 611:1214–7. 10.53738/REVMED.2018.14.611.1214 29944278

[18] Stabilini C van Veenendaal N Aasvang E Agresta F Aufenacker T Berrevoet F. Update of the international HerniaSurge guidelines for groin hernia management. BJS Open. (2023) 7(5):zrad080. 10.1093/bjsopen/zrad080 37862616 PMC10588975

[19] Traoré D Diarra L Coulibaly B Bengaly B Togola B Traoré A. Hernie inguinale en afrique subsaharienne: quelle place pour la technique de shouldice? Pan Afr Med J. (2015) 22:50. 10.11604/pamj.2015.22.50.6803 26664551 PMC4662532

[20] Chendjou WT Christie SA Carvalho M Nana T Wepngong E Dickson D. The prevalence and characteristics of untreated hernias in southwest Cameroon. J Surg Res. (2019) 244:181–8. 10.1016/j.jss.2019.06.035 31299434

[21] Ohene-Yeboah M Abantanga FA. Inguinal hernia disease in Africa: a common but neglected surgical condition. West Afr J Med. (2011) 30(2):77–83. 21984452

[22] Sine B Ndong A Sarr A Diallo A Faye M Bagayogo N. Prise en charge des hernies inguinales dans un service d'urologie d'afrique subsaharienne: à propos de 205 CAS. J Afr Chir Digest. (2019) 19:2805–8.

[23] Valentin V Mahamat MA Siyangar NA Michael TO Barthelemy V Koldimadji M. Simultaneous management of inguinal hernia and benign prostatic hypertrophy in a single operation at the chu D'abeche/Chad. Open Journal of Urology. (2023) 13(9):363–70. 10.4236/oju.2023.139041

[24] Granados EA Villavicencio H Sole-Balcells F. Should hernioplasty be combined with surgery of the prostate? Arch Esp Urol. (1998) 51(8):767–70. 9859581

[25] Hsu TW Tseng WH Huang SK Chiu AW Li CF Shiue YL. Transurethral resection of the prostate (TURP) and concomitant inguinal hernioplasty: a single-center experience. BMC Urol. (2024) 24:188. 10.1186/s12894-024-01571-z 39217318 PMC11365149

[26] Ndong A Sine B Faye M Diallo AC Bagayogo NA Ndiaye M. Particularités diagnostiques, thérapeutiques et évolutives des hernies inguinales du sujet de plus de 60 ans. PAMJ-CM. (2020) 2:159. 10.11604/pamj-cm.2020.2.159.22322

[27] Osifo O Amusan TI. Outcomes of giant inguinoscrotal hernia repair with local lidocaine anesthesia. Saudi Med J. (2010) 31(1):53–8. 10.15537/1658-3175.4932 20062900

[28] Olaogun JG Afolayan JM Areo PO Ige JT. Repair of groin hernia under local anaesthesia in secondary health facility. ANZ J Surg. (2018) 88(4):E294–7. 10.1111/ans.13849 27925429

[29] Argo M Favela J Phung T Huerta S Local VS. Other forms of anesthesia for open inguinal hernia repair: a meta-analysis of randomized controlled trials. Am J Surg. (2019) 218(5):1008–15. 10.1016/j.amjsurg.2019.06.024 31288925

[30] Ndong A Diallo AC Falola A Ndiaye MA Faye M Faye PM. Choice of surgical technique in groin hernia surgery among residents in Senegal: experience and influencing factors. J Abdom Wall Surg. (2025) 4:14076. 10.3389/jaws.2025.14076 40520118 PMC12162351

[31] Wilhelm TJ Anemana S Kyamanywa P Rennie J Post S Freudenberg S. Anaesthesia for elective inguinal hernia repair in rural Ghana—appeal for local anaesthesia in resource-poor countries. Trop Doct. (2006) 36(3):147–9. 10.1258/004947506777978046 16884617

[32] Warwick A Oppong C Boateng Doah B Kingsnorth A. Inguinal Hernia Repair is Safe in Africa (2013) Available online at: https://www.ajol.info/index.php/ecajs/article/view/97301 (accessed February 04, 2026).

[33] Ndong A Tendeng JN Diallo AC Diao ML Diop S Dia DA. Is desarda technique suitable to emergency inguinal hernia surgery? A systematic review and meta-analysis. Annals of Medicine and Surgery. (2020) 60:664–8. 10.1016/j.amsu.2020.11.086 33312559 PMC7721661

[34] Coco D Leanza S Reina GA. Use of the desarda technique in emergency settings: a comprehensive review. Maedica (Buchar). (2022) 17(2):481–6. 10.26574/maedica.2022.17.2.481 PMC9375861 36032629

[35] Philipp M Leuchter M Lorenz R Grambow E Schafmayer C Wiessner R. Quality of life after desarda technique for inguinal hernia repair-A comparative retrospective multicenter study of 120 patients. J Clin Med. (2023) 12(3):1001. 10.3390/jcm12031001 36769652 PMC9917682

[36] Faylona JM. Evolution of ventral hernia repair. Asian J Endosc Surg. (2017) 10(3):252–8. 10.1111/ases.12392 28631265

[37] Francis B Mubezi I Kakande I Oppong C Akoh J Tatumwa BD. Postgraduate surgical hernia training with a standardized hernia surgery training tool: training while serving humanity. Ann Med Surg (Lond). (2025) 87(12):8304–8. 10.1097/MS9.0000000000004183 41377261 PMC12689123

